# Harris’ Dictionary of Dental Science

**Published:** 1849-07

**Authors:** 


### HARRIS' DICTIONARY OF DENTAL SCIENCE.

This work, so important to every dental practitioner, and without which his library could not be perfected, is just from the press of the Messrs. Lindsay & Blackiston, Philadelphia. The work comprises 780 pages, royal octavo, is gotten up on good paper, clear type, and well bound; and in artistic execution, will not detract from the well earned reputation of the publishers. As an addition to the literature of our profession, every Dentist will, we feel assured, hail it as a work just suited to the wants of a distinct department of medical science; and which places in advance one step further, dental science side by side with the other specialities of a beneficent and enlightened profession.

Every practitioner of Dentistry must have often been forcibly struck with the vast field through which he would often have to explore, to collect such *items* of intelligence as he might wish to put to a practical test at the time. Works on Pathology, Therapeutics, Anatomy, Chemistry, Pharmacy, and our best Lexicons only here and there giving a hint on the subject, the whole would have to be conned, digested and arranged, before it could be used. This has been one of the great difficulties in giving a definite form and character to dentistry as a science. The work under consideration has done more to remove the difficulty than any other ever published, It is a compendium of facts which lay scattered throughout the vast field of medical and dental science, arranged, simplified, and placed at hand for practical use. A mere Dictionary of Medical and Dental terms used in the profession, although vastly needed, yet would not have filled so entire that void as the one just published, which embraces Biography, Bibliography, and Medical terminology. It brings up the history of the science to the present time, and forms a library in and of itself, far more perfect than is usually met with among half those claiming to be Dentists. To the Physician we think the work will commend itself, for while he meets with much that is truly in the *line* of his profession, he at the same time gets in a condensed form, a correct history of a speciality of his profession, the entire neglect of which would render his treatment of many aggravated diseases, utterly inefficient.

We are aware that the preparation of such a work cost a great deal of most persevering labor, and must have required at the hands of Prof. Harris, a rigid and economical distribution of his time, to have so well accomplished the task. We hope all this will be duly appreciated by the Profession, and that an extensive sale of the work will fully remunerate him for his arduous labor.

We hope, in conclusion, we shall not be thought censorious, if we allude to one or two errors, as we think, in arrangement, especially as it regards the history of the profession in the United States. It would have been more in accordance with the design of the work, had each association and institution been placed in its appropriate alphabetical order, and treated of separately. A distinct history of the gradual development of the profession, would thus have been preserved and arranged. We hope that this will be obviated when another edition is published, and the Biographical department be also enlarged. We however, think much, perhaps more than could have been expected has been accomplished.—[Cin. Ed.

